# Metabolic bone health considerations in giant cell arteritis and polymyalgia rheumatica

**DOI:** 10.1177/17455057221147385

**Published:** 2023-01-10

**Authors:** Candice Low, Richard Conway

**Affiliations:** 1 Department of Rheumatology, St. James’s Hospital, Dublin, Ireland; 2 Department of Clinical Medicine, Trinity College Dublin, Dublin, Ireland

**Keywords:** fracture, giant cell arteritis, glucocorticoids, osteoporosis, polymyalgia rheumatica, steroids

## Abstract

Giant cell arteritis (GCA) and polymyalgia rheumatica (PMR) are two common systemic inflammatory conditions with a combined lifetime risk of approximately 3.5% in women and 1.5% in men. They are intimately associated with the aging process, virtually never occurring prior to 50 years of age and becoming more common over time. The reasons for this are unclear, but likely relate in part to factors related to aging of the immune system. The treatment of both GCA and PMR is traditionally based on glucocorticoids, frequently requiring a prolonged treatment course over long periods of time. Other medications are belatedly entering our treatment armamentarium, but their exact place in treatment algorithms remains to be fully defined and it is likely glucocorticoids will remain a cornerstone of our treatment in GCA and PMR for the foreseeable future. As a result, people with GCA and PMR will continue to be exposed to a significant cumulative glucocorticoid burden with all of the attendant potential adverse events, including osteoporosis. The predominantly post-menopausal female population that most commonly develops PMR and GCA is also the population that is most affected by osteoporosis. Given the risk of glucocorticoid-induced osteoporosis and subsequent fragility fractures, a planned treatment approach from glucocorticoid initiation is needed in these conditions. For the majority of patients, this will entail ensuring sufficiency of calcium and vitamin D as well as antiresorptive treatments. In this article, we discuss considerations around optimisation of metabolic bone health in GCA and PMR.

## Introduction

Giant cell arteritis (GCA) and polymyalgia rheumatica (PMR) are two systemic inflammatory conditions that demonstrate a striking age association. Both conditions are hardly ever seen prior to 50 years of age and become more common with increasing age until a plateau in the mid-70s.^1–3^ While still contentious, it is hypothesized that GCA and PMR represent two extremes of a single disease spectrum, with GCA characterized by vascular involvement and PMR by periarticular disease.^4,5^ This hypothesis is supported by shared disease mechanisms, demographic features, and clinical response to certain treatments.^2,4,6–8^ Due to this association with increasing age, the two are frequently associated with other comorbidities associated with increasing age, including osteoporosis.^9,10^ This is further complicated as both the inflammatory process of the diseases themselves and the negative consequences of their treatment can be associated with de novo onset or worsening of bone health issues.^11^ Too rigid a focus on the highly symptomatic inflammatory aspects of these rheumatic diseases frequently lead to these secondary consequences of initially silent complications such as osteoporosis being overlooked, at least until fracture ensues.^12^ This situation is further exacerbated by a lack of ownership of these conditions; both the treating rheumatologist and primary care physician may defer responsibility to the other party with the end result that no one ends up managing this important aspect of the disease. Appropriate management of osteoporosis risk in GCA and PMR is crucial to minimize subsequent incident fractures.

## Giant cell arteritis

GCA is a large vessel vasculitis. It is the prototypical large vessel vasculitis in the older population, in contrast to Takayasu’s arteritis which affects the younger population. GCA demonstrates one of the strongest age predilections of any rheumatic disease, with a peak incidence in the mid-70s age range. Another curious aspect of the epidemiology of GCA is that it demonstrates a marked geographical variation, being more common in Northern European populations.^13^ This striking variation in distribution in different populations and geographic regions is of a strength not usually seen outside of infectious disease. This led to multiple attempts to determine the infectious aetiology behind GCA.^14–16^ However, further examination has suggested that this seems to be a genetic association rather than an environmental or infectious factor in these regions as immigrant populations of predominantly Northern European extraction (e.g. Olmstead County) appear to maintain the risk of their area of origin.^1^ While often considered a rare disease, this is not true. While what constitutes rarity is relative and subjective, the lifetime risk of GCA of 1% for women in such populations is unlikely to qualify as such.^1^ GCA is twice as common in women compared with men.^1^ GCA is potentially a severe disease with the occurrence of permanent visual loss or stroke in a substantial minority of patients at disease onset.^17,18^ In the longer term, large vessel aneurysmal disease is a significant concern.^19,20^ This particularly affects the thoracic aorta and can lead to aneurysmal rupture and sudden death. However, in clinical practice it is often glucocorticoid-related adverse events that are the greatest and most pervasive challenge in people with GCA.^9^ The treatment of GCA in terms of both chronology and the individual patient is characterized by early treatment successes followed by later difficulties and complications. Glucocorticoids were introduced to the treatment paradigm in GCA nearly 70 years ago and remain a currently indispensable cornerstone of treatment. For many years, multiple other agents were found to have either a limited benefit, been ineffectual, or even paradoxically harmful.^21–23^ For the individual patient, the satisfaction of dramatic early treatment responses is tempered by later treatment-related adverse events.^9,12,24^ We now have a proven effective adjunctive treatment to glucocorticoids in GCA in tocilizumab; a monoclonal antibody to the IL-6 receptor.^6,25^ The phase 3 GiACTA randomized controlled trial including 250 patients split 50/50 between new onset and relapsing Giant-Cell Arteritis Actemra (GCA). Tocilizumab-treated patients demonstrated increased rates of sustained remission (53%–56% vs. 14%–18%) at 52 weeks and a 50% reduction in the cumulative glucocorticoid dose (1862 mg vs. 3818 mg). Serious adverse events were also less frequent in the tocilizumab treated patients (14%–15% vs. 22%–25%).^6^ This is supported by a number of observational studies demonstrating a substantial real-world effect of tocilizumab on reducing cumulative glucocorticoid doses.^26,27^ There are a number of other agents in advanced stages of development for GCA including mavrilimumab, secukinumab, and janus kinase inhibitors.^28^ The exact position of all of these agents in the GCA treatment paradigm remains somewhat uncertain and is subject to jurisdictional financial controls and other measures in addition.

## Polymyalgia rheumatica

PMR is an inflammatory rheumatic condition that is as common as rheumatoid arthritis but less well recognized, at least among the general population.^1^ Like GCA, it shows a marked geographic variation, being more common in Northern European populations. Women are affected 2 to 3 times as commonly as men. PMR is almost unheard of in those less than 50 years of age and becomes more common with further increasing age. PMR is a disease whose chief presenting features are proximal limb girdle aching and stiffness, accompanied by raised inflammatory markers. PMR classically has a pronounced diurnal variation manifested by marked early morning stiffness. The clinical manifestations of PMR appear to result predominantly from extracapsular disease around the proximal limb joints in contrast to the joint-based disease seen in rheumatoid arthritis.^29,30^ PMR and GCA appear to be on the same spectrum of disease, and both clinical and experimental evidence suggests that PMR may represent a *forme fruste* of GCA.^4,5^ Approximately 50% of patients with GCA either have or subsequently develop PMR and 11%–20% of patients initially presenting with PMR subsequently develop GCA.^31–34^ GCA may be occult in patients presenting with PMR; in up to 30% of patients presenting with symptoms consistent with isolated PMR, imaging studies demonstrate involvement of the proximal limb arteries consistent with GCA, in addition to periarticular uptake consistent with PMR.^5^ Like GCA, PMR is predominantly treated with glucocorticoids, albeit at lower doses. Response to even relatively low doses of glucocorticoids is characteristically dramatic and rapid in PMR. In the last year, clinical trials have demonstrated the efficacy of tocilizumab and, more surprisingly, rituximab in PMR.^7,35^

## Reasons behind age relationship

The factors underlying the striking age predilection for both GCA and PMR remain uncertain. A commonly proposed factor is that of immunosenescence.^36^ Immunosenescence is most often used to refer to the degeneration of the immune system with aging resulting in a reduced protective response to infections and reduced reactivity to vaccinations. The oft overlooked other aspect of this physiologic Janus is that the aging immune system also becomes more predisposed to autoreactivity, both through a pro-inflammatory disposition and an increasing tendency to confuse host autoantigens for pathogens. The term ‘inflamm-aging’ has been coined for this process.^37^ This immune aging process appears to begin from the third decade of life but rapidly accelerates from 50 years onwards, consistent with the age distribution of PMR and GCA.^38^ Age-related decompensation of the normal CD8+ T-cell population facilitating unregulated activation of CD4+ effector T cells and loss of efficiency of checkpoint mechanisms have all been shown to play a role in the aging immune system in GCA.^38^ While substantial steady incremental progress has been made by the hard work of dedicated researchers in this area, the pathogenesis of GCA and PMR continues to be a challenging and ongoing open field of research efforts.

## Glucocorticoid effect on bone

Glucocorticoids are an indispensable component for the optimum functioning and physiology of the human body.^39^ Conceptually, many glucocorticoid-related adverse events can be understood of as an amplification of the normal physiologic roles and effects of glucocorticoids.^39^ In terms of bone physiology, glucocorticoids induce an early high bone turnover state.^40^ The consequences of this can be rapid and profound, with increased fracture risk becoming evident within the first 3 months of glucocorticoid initiation.^41^ With continuation of administration of glucocorticoids over a more prolonged timeframe, there is a transition to a low bone turnover state with a net loss of bone strength due to a more pronounced impact on synthesis than on resorption. The peak loss of bone occurs within the initial 3–6 months of glucocorticoid use; however, there continues to be an ongoing accumulation of bone loss with continuation of glucocorticoids on a more prolonged basis.^41^ Both higher daily glucocorticoid dose and higher cumulative glucocorticoid dose are associated with a progressive increase in fracture risk.^42,43^ Even low doses of glucocorticoids are associated with increased fracture risk, particularly when given for prolonged durations.^44^

## Glucocorticoid use in GCA and PMR

**Figure 1.**
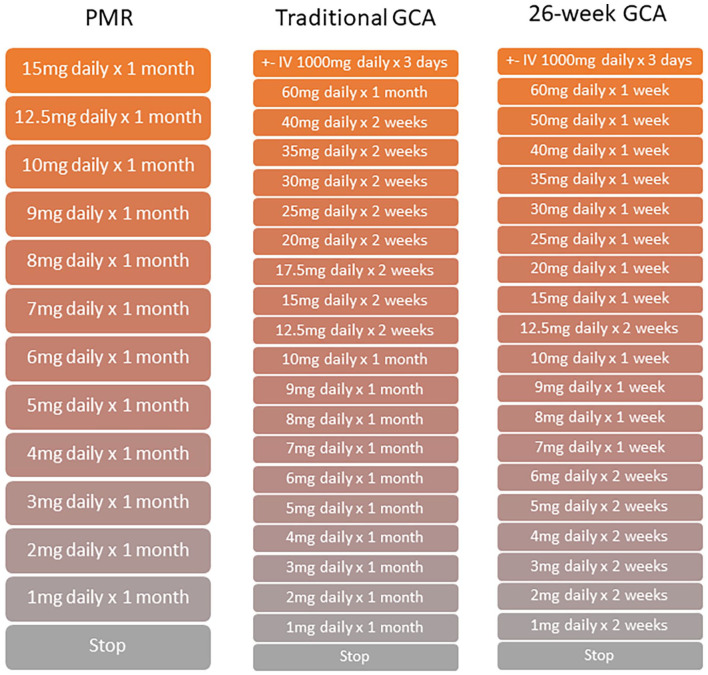
Glucocorticoid treatment regimens in PMR and GCA.

Prior to consideration of mitigation strategies, it is illustrative to consider the often underappreciated scale of glucocorticoid use in PMR and GCA. Initial doses of prednisolone of 15 mg daily and 40–60 mg daily, respectively, do not reflect the full impact.^45–47^ The major consideration is the duration of therapy when glucocorticoids are used as monotherapy, with even patients with successful tapering without relapse generally experiencing 18 months of glucocorticoid treatment in GCA and 12 months in PMR.^45–48^ This is demonstrated by the cumulative glucocorticoid burden experienced. Even with the 52-week glucocorticoid taper in GiACTA, which is more rapid than standard clinical practice, this results in almost 4000 mg of glucocorticoid over that year.^6^ Illustrative examples of steroid tapering regimens used in clinical practice in GCA and PMR are shown in Figure 1.^45,47,48^

## Osteoporosis and fracture rates in PMR and GCA

A number of studies have evaluated rates of osteoporosis and fragility fracture in PMR and GCA. Not surprisingly they are high. A UK-based database study of 15,000 individuals reported fracture rates 63% and 67% higher for PMR and GCA, respectively, with less than 13% of patients receiving bisphosphonates.^49^ Another set of linked articles using the same dataset looked at 5000 people with GCA and demonstrated incidence rate ratios of 2.4 for osteoporosis and 1.4 for fracture in GCA; multivariate modelling demonstrated odds ratios of 1.9 for osteoporosis and 2.6 for fracture in GCA compared with the general population.^50,51^ Two French studies reported fracture rates of 13% and 9% in GCA patients.^52,53^ In the Vasculitis Clinical Research Consortium prospective multicentre longitudinal GCA cohort, 22 of 204 patients developed new osteoporosis over 3.5 years of follow-up, in addition to 16 of 204 who had osteoporosis at baseline.^54^ A population-based study of 768 GCA patients from the Skåne region in Southern Sweden showed a relative risk of 2.81 for osteoporosis in GCA compared with an age- and sex-matched population.^55^ A Korean study reported 17 fractures in 12 of 95 PMR patients over 433 person years of observation.^56^ A UK-based cohort study of 652 people with PMR reported 72 fractures at month 12 and 62 at month 24; concerningly less than 50% of patients were prescribed any form of treatment (including calcium/vitamin D) in this study.^57^ An Italian study followed 222 PMR patients over a mean duration of 60 months; 55 developed osteoporosis and 31 had fragility fractures. Glucocorticoid dose and duration were significantly associated with osteoporosis and fracture rates.^58^

## Assessing and minimizing fracture risk

A key concept to remember is that the clinically important end point in osteoporosis is fracture. Osteoporosis is an asymptomatic disease until fracture occurs. The numbers on the DXA scan are irrelevant other than their implications for future fracture risk. The management of osteoporosis frequently focuses on medication strategies to increase bone mineral density. It is easy to fall into the trap of focusing too much on the T-score while ignoring other potentially more important clinical factors. While osteoporotic fragility fractures frequently occur with minimal or no trauma, falls remain an important and unaddressed causative factor in many fractures, particularly hip and distal radius fractures.^59^ Exercise programmes targeted at muscle strengthening and improving balance are beneficial.^60^ Strategies aimed at ameliorating environmental and medication risk factors for falls may have immediate benefits in the reduction of falls and subsequent fractures.^61^ Addressing other risk factors for bone loss, including smoking, alcohol, and the appropriate management of other active contributory medical conditions, are important adjunctive measures in overall osteoporosis management.^61^

While glucocorticoids do reduce bone mineral density as measured by DXA, they also increase fracture risk independent of reductions in bone mineral density.^62^ Therefore, at any given bone mineral density, the fracture risk is increased in individuals receiving glucocorticoids.^62^ This is reflected in the incorporation of glucocorticoids in the FRAX fracture risk prediction model.^63^

**Figure 2.**
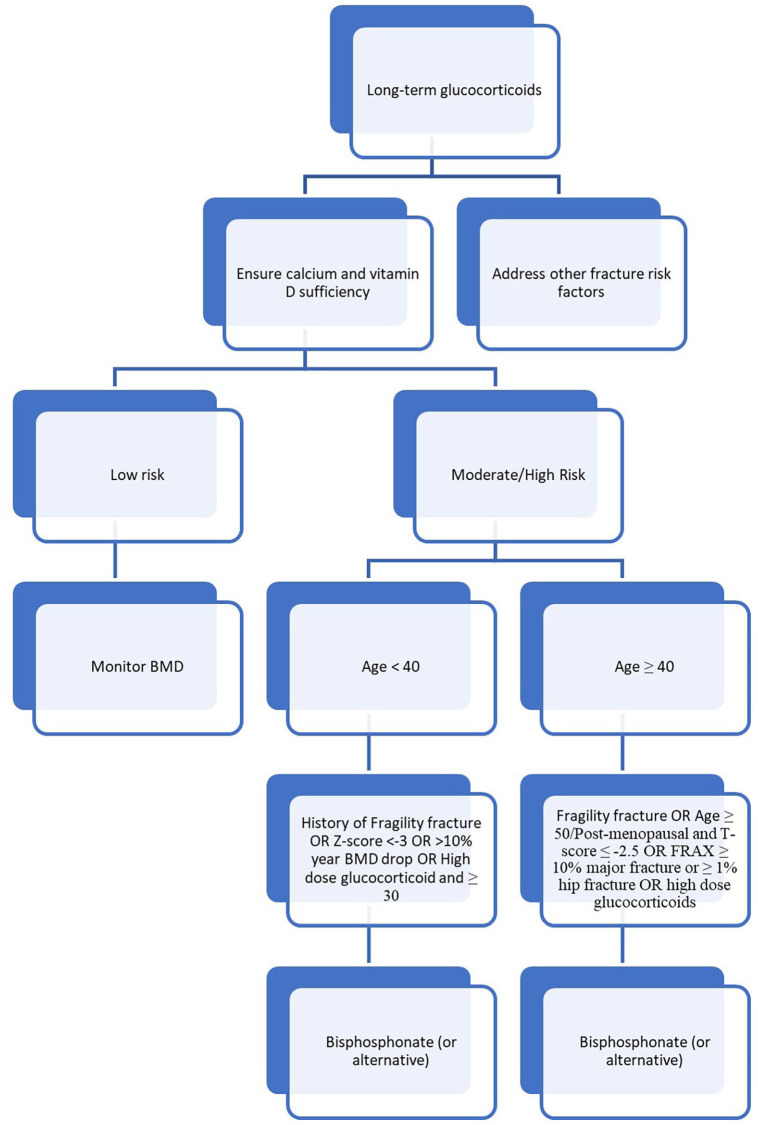
Prevention of glucocorticoid-induced osteoporosis.

**Figure 3.**
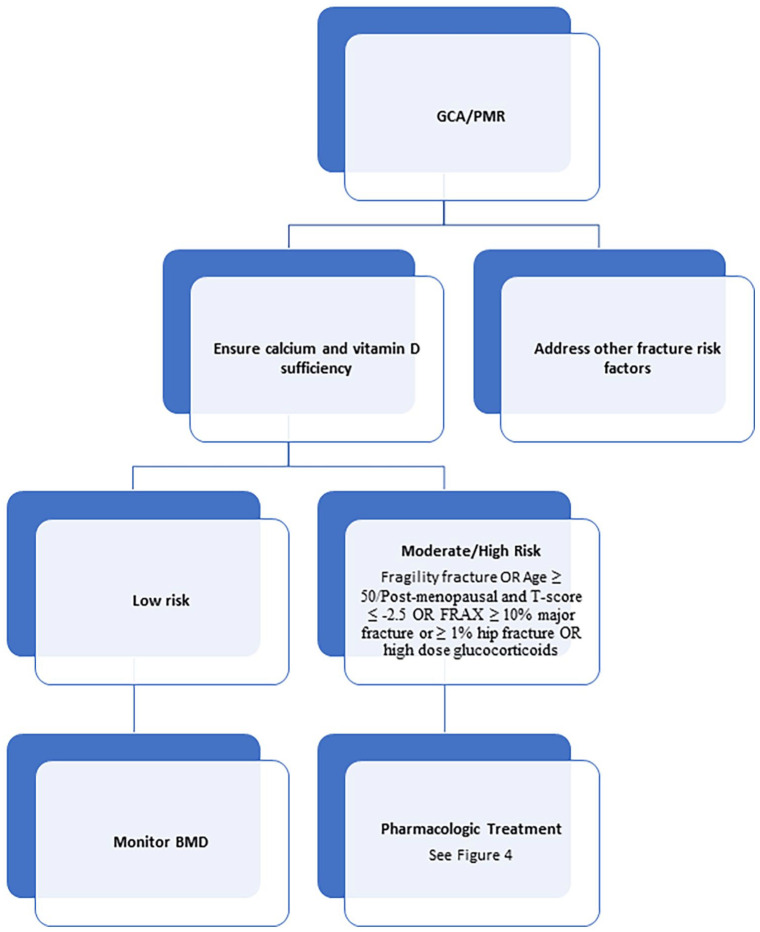
Prevention of glucocorticoid-induced osteoporosis in PMR and GCA.

All patients with PMR and GCA should have a DXA scan performed.^47,64^ A basic tenet of the management strategy when considering glucocorticoid-induced osteoporosis is that it is far easier to prevent bone loss in the first place than it is to rebuild it following such loss. While not all patients on glucocorticoids require pharmacologic treatment, many do.^64^ This is particularly true for those who have other risk factors for fracture common to GCA and PMR, including increasing age.^65^ This risk stratification in glucocorticoid-induced osteoporosis in general is complex and individualized (Figure 2).^64^ As almost all patients with GCA/PMR are ⩾50 years old, this algorithm can be simplified (Figure 3).

Patients with identified established osteoporosis (history of fragility fracture or BMD T-score ⩽−2.5) are at greatest risk of fracture and should have pharmacologic treatment prescribed. In those without established osteoporosis, a clinical fracture risk assessment should be performed, whether using a formalized tool such as FRAX^®^ or using clinician gestalt.^64^ FRAX^®^ is the most widely used fracture risk prediction model, although many other models such as Q-fracture and Garvan also exist.^66^ FRAX^®^ utilizes a defined set of factors to predict 10-year fracture risk in individuals aged 40–90 years who have not previously been treated with pharmacologic therapy for osteoporosis. Importantly in the setting of glucocorticoid-induced osteoporosis FRAX^®^ does not take into account glucocorticoid dose or duration, and therefore these crucial fracture predictive factors must be added to consideration of overall fracture risk.^67^ One suggested approach is that for individuals taking >7.5 mg prednisolone equivalent a day to increase the risk prediction for major osteoporotic fracture by 15% and for hip fracture by 20%.^67^ FRAX^®^ subsequently stratifies individuals as being at low, moderate, or high risk of fracture based on region-specific thresholds. For the purposes of pharmacologic treatment decisions in glucocorticoid-induced osteoporosis, patients at moderate and high risk are generally grouped together. The treatment thresholds for North America for moderate/high risk are a 10-year probability of major osteoporotic fracture of ⩾10% and/or a 10-year probability of hip fracture of ⩾1%.^64^ Those identified as being at moderate or high risk of fracture should have pharmacologic treatment irrespective of overall DXA results.^64^

## Bone health medication in GCA and PMR

### Getting the building blocks right

Bone is a complex biomechanical organ with a vital structural role. Similar to the construction of any structure, it is necessary to have the appropriate raw materials to ensure structural integrity and longevity. The crucial importance of calcium and vitamin D to bone health has long been emphasized.^68^ However, it is sagacious to remember that too much of a good thing can also be harmful; and it is perhaps more appropriate to frame this as ensuring calcium and vitamin D sufficiency rather than supplementing these blindly.^69^ Excess calcium and vitamin D have been associated with an increased risk of cardiovascular disease and with more frequent ureteric stones, respectively.^70,71^ While the magnitude of this risk is small and somewhat controversial, if there is no benefit to oversupplementation then it should be avoided. As a mentor of one of the authors (RC) once stated it, ‘calcium supplementation is for people with calcium deficiency and vitamin D supplementation is for people with vitamin D deficiency’ (Carey JJ, personal communication). While this assessment is frequently neglected in practice, it is relatively easily performed, and at least for vitamin D, relatively accurate. Serum 25-hydroxyvitamin D levels, with some caveats, reflect vitamin D sufficiency and are easily measured on a blood draw.^72^ Calcium is significantly more complex, in that the relationship of serum calcium to adequate bone calcium is almost non-existent except in extreme circumstances. Human physiology, particularly cardiac and muscle function, is exquisitely sensitive to perturbations in serum calcium; therefore, the body will sacrifice any other site of calcium, particularly bone, to maintain calcium homeostasis in the blood and tissues. Dietary calcium intake questionnaires represent the best available means to assess long-term calcium sufficiency.^68^ If dietary deficiency is identified, increasing calcium intake is warranted. However, this should not result in a knee jerk reaction to prescribe a supplement. Dietary modification with an increased intake of calcium-rich foods is preferable in terms of tolerability and potentially safety.^68,73,74^ Only in the event that this is not possible or practical to ensure sufficiency through diet is supplements warranted.^68,75^ Vitamin D insufficiency is very common, especially in parts of the world not blessed with copious sunlight, which is the main source of vitamin D.^76,77^ Physiologic vitamin D production is also dependent on skin exposure to sunlight, so those living even in sunnier climes who spend their time indoors or with their skin covered are also vulnerable.^78^ Increasing exposure of the skin to sun is generally not medically advisable due to the association with sun damage and later skin cancer.^79^ Dietary sources are generally not sufficient.^80^ Vitamin D deficiency is therefore generally most appropriately managed by supplementation.^61^ This may be achievable with vitamin D supplemented milk products, which may be sufficient to avoid a separate oral vitamin D supplement.^81^ There are two formulations of vitamin D available as supplements: ergocalciferol (vitamin D2) and cholecalciferol (vitamin D3). Some studies suggest cholecalciferol increases serum 25-hydroxyvitamin D more efficiently than ergocalciferol, and therefore we generally prefer cholecalciferol when available.^82,83^ There are two approaches for vitamin D repletion. One approach is to start a 600-1000 IU vitamin D supplement and increase serum 25-hydroxyvitamin D slowly to sufficiency.^72,84^ Another approach is a loading dose approach, with an initial loading dose of 50,000 IU weekly for the first 6–8 weeks and then a maintenance dose of 600–1000 IU.^72,84^ Whichever approach is used, a subsequent recheck of serum 25-hydroxyvitamin D to ensure the success of supplementation leading to repletion is essential.^84^ Adequate dietary protein intake is also crucial in maintaining bone health, with recent data emphasizing the importance of sufficient protein consumption in preventing falls and fractures.^85^

### Affecting bone remodelling

When considering which medications to use, an important consideration is that it is likely for most patients with GCA and PMR that they will not be on glucocorticoids long-term.^45,47,64^ Therefore, an agent which is effective while being taken and with no association with rebound bone loss on cessation is most attractive.^86^

**Figure 4.**
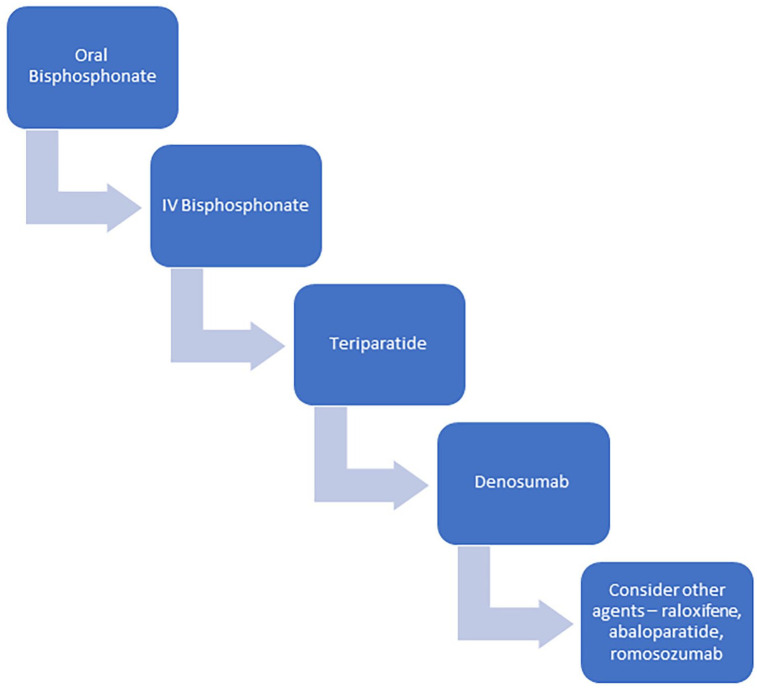
Treatment algorithm for osteoporosis in PMR and GCA.

There are three main classes of pharmacologic agents utilized in glucocorticoid-induced osteoporosis: the antiresorptive agents bisphosphonates and denosumab, and the anabolic agent teriparatide.^64^ A suggested treatment algorithm is shown in Figure 4.

Bisphosphonates are favoured in the setting of expected temporary use for several reasons, especially due to their prolonged beneficial effects after stopping the agent due to their incorporation in bone.^64^ Essentially this means, that in individuals who do not have co-existent osteoporosis for other reasons, that the majority of the time, the bisphosphonate can be stopped when the glucocorticoids are.^64^ Similarly, as the duration of steroid use will often be 1–2 years, an oral bisphosphonate rather than an annual intravenous infusion is usually more pragmatic.

Teriparatide is a daily injection for 2 years and should be followed by an antiresorptive agent in order to preserve BMD gains. There are theoretical advantages to teriparatide over bisphosphonates in the setting of glucocorticoid-induced osteoporosis. The predominant long-term skeletal effect of glucocorticoids is to reduce bone formation and bisphosphonates as purely antiresorptive agents do not address this mechanism of bone loss.^87^ However, clinical evidence with fracture endpoints to support this approach is limited, and in the setting of non-permanent glucocorticoid use, its daily subcutaneous administration and cost make teriparatide a less attractive option.^64^ In the setting of established osteoporosis with factors in which teriparatide would normally be the treatment of choice, then it remains appropriate to utilize in the setting of glucocorticoid use.

Denosumab, which inhibits RANK-ligand, has a particular concern with its use, in that it has a rapid off effect once ceased with an associated precipitous drop in bone mineral density.^86^ This is the reason that drug holidays are not appropriate in individuals receiving denosumab, and is also a concern around the potential short-term prophylactic use in the setting of glucocorticoids, although data here are limited.^86^ We prefer not to use denosumab in this setting unless there is pre-existing established osteoporosis which would justify long-term denosumab.

Abaloparatide is a synthetic analogue of parathyroid hormone-related protein (PTHrP). In post-menopausal osteoporosis, it is generally viewed as equivalent to teriparatide.^88^ However, it has not been specifically evaluated in glucocorticoid-induced osteoporosis, and we prefer the use of teriparatide in this setting. Romosozumab, an anabolic sclerostin inhibitor has been approved for use in post-menopausal osteoporosis but has not been studied in glucocorticoid-induced osteoporosis, and we therefore recommend against its use in this setting, unless no alternative agents are available.^89^ Selective-oestrogen receptor modulators such as raloxifene are potential alternatives when other agents are not suitable but are not preferred as they have lower antifracture efficacy than other available agents.^90^

### Determining the Need for continuing treatment

As a general rule, most individuals need to continue their bone prophylaxis as long as they remain on glucocorticoids, and certainly as long as they are >5 mg/day prednisolone equivalent dose.^64^ Osteoporosis is incredibly common with half of women and one quarter of men suffering an osteoporotic fracture during their lifetime.^91^ Osteoporosis is also very much a hidden disease with the majority of individuals undiagnosed until they suffer the end consequence of a fracture. Many individuals undergoing glucocorticoid treatment meet traditional screening criteria for DXA even prior to glucocorticoids, but even in the absence of this should undergo bone density assessment prior to consideration of cessation of bone prophylaxis.^47,64^ Due to both the high baseline prevalence of osteoporosis and the deleterious effects of glucocorticoids (which are limited but not fully prevented by prophylaxis), many individuals will warrant continuation of osteoporosis treatment following glucocorticoid cessation.^64^ These people should then undergo ongoing care with similar strategies to any other individuals with osteoporosis, while remaining cognisant that disease relapse may necessitate further glucocorticoid use in the future.^64^

### Role of early initiation of glucocorticoid sparing therapy

The addition of a second agent to glucocorticoid monotherapy has been demonstrated to significantly reduce cumulative glucocorticoid dose in both GCA and PMR.^6,7,35^ As cumulative glucocorticoid dose is intimately associated with rates and severity of adverse events, it would appear a prudent consideration as to whether to target individuals at high risk of glucocorticoid toxicity for initial use of these agents. This is both easier to justify and supported by stronger data in GCA, where the cumulative glucocorticoid dose is significantly higher, than in PMR.^6^ Increasingly both guidelines and real-world practice are leading to increased upfront use of tocilizumab in newly diagnosed patients, particularly those at higher risk of glucocorticoid-related adverse events, in addition to its clear role in relapsing disease.^45,48^ This of course is dependent on funding mandates in individual health care systems.^92^

### Limitations of current review

The current review is a narrative synthesis of the literature and as such is based on the opinions and clinical experience of the authors rather than being purely evidence based. The scientific basis for many aspects of glucocorticoid-induced osteoporosis in general, and particularly with regard to the specifics of the management of glucocorticoid-induced osteoporosis in GCA and PMR, is generally supported by a weak level of evidence and so should be viewed with cognisance of these limitations. Particularly, even though the initial and cumulative glucocorticoid doses differ significantly in GCA and PMR, there is no clear evidence to support a different approach to the prevention and management of glucocorticoid-induced osteoporosis in these two diseases.

### Summary

PMR and GCA are relatively common chronic conditions with traditionally high cumulative glucocorticoid burdens. As a result, glucocorticoid-induced osteoporosis and subsequent fracture is common in this patient population and associated with considerable morbidity and mortality. Judicious use of existing treatment options can ameliorate these risks to a considerable extent and should be employed. The era of steroid-sparing adjuvant treatments in GCA and PMR is upon us and may have additional benefits of improved bone health outcomes.

## Declarations

**Ethics approval and consent to participate:** Not applicable.

**Consent for publication:** Not applicable.

**Availability of data and materials:** No additional material exists.
